# Backdoor Training Paradigm in Generative Adversarial Networks

**DOI:** 10.3390/e27030283

**Published:** 2025-03-09

**Authors:** Huangji Wang, Fan Cheng

**Affiliations:** Department of Computer Science and Engineering, Shanghai Jiao Tong University, Shanghai 200240, China; wanghuangji@sjtu.edu.cn

**Keywords:** backdoor attack, generative model, diffusion model, GAN, paradigm

## Abstract

Backdoor attacks remain a critical area of focus in machine learning research, with one prominent approach being the introduction of backdoor training injection mechanisms. These mechanisms embed backdoor triggers into the training process, enabling the model to recognize specific trigger inputs and produce predefined outputs post-training. In this paper, we identify a unifying pattern across existing backdoor injection methods in generative models and propose a novel backdoor training injection paradigm. This paradigm leverages a unified loss function design to facilitate backdoor injection across diverse generative models. We demonstrate the effectiveness and generalizability of this paradigm through experiments on generative adversarial networks (GANs) and Diffusion Models. Our experimental results on GANs confirm that the proposed method successfully embeds backdoor triggers, enhancing the model’s security and robustness. This work provides a new perspective and methodological framework for backdoor injection in generative models, making a significant contribution toward improving the safety and reliability of these models.

## 1. Introduction

With the rapid development of deep learning, powerful models have emerged for learning complex data such as high-dimensional data, temporal data, spatial data, and graph data. Generative models are a class of powerful models that aim to learn the distribution of data in order to generate new samples that resemble real data [1]. Common types of generative models include generative adversarial networks (GANs) [2,3,4,5,6,7], variational autoencoders (VAEs) [8,9,10,11], diffusion models [12,13,14,15], and autoregressive models [16]. These models have found widespread application in multimodal generation tasks [17,18,19,20,21,22]. Despite their significant success, generative models face several security and privacy challenges, one of which is the threat of backdoor training attacks. These attacks raise concerns about the security of generative models in safety-critical scenarios, such as privacy protection [23,24], copyright claims [25,26], and model integrity [27].

However, state-of-the-art deep neural network models consolidate the knowledge of researchers while consuming vast amounts of data and computational resources, leading to high costs. Although models as a product for commercial sale (Model as a Service, MLaaS) [28] can be a lucrative business model, the low cost of stealing, copying, or misusing these models poses significant risks.

Backdoor training, a type of backdoor attack, or a type of ownership verification, involves injecting a trigger into a small subset of training data to implant a backdoor into the model [29]. The core goal of this method is to ensure that the model performs normally on regular inputs while producing a pre-determined output under specific trigger conditions. Related works [30,31,32,33] indicate that this type of attack poses a serious threat to deep neural network-based models, as backdoor triggers are relatively easy to implant but difficult to detect or remove [34], which means this is also a method to protect the ownership. A key feature of backdoor attacks is that they do not degrade the model’s performance on clean test inputs, yet they allow the attacker to control the model’s behavior for any test input containing the backdoor trigger. This makes it challenging to detect such attacks based solely on the model’s performance on clean test sets [35,36,37,38].

While model ownership protection and watermarking have been explored in generative models to ensure intellectual property rights, these approaches [39,40,41,42,43,44,45,46] focus on embedding identifiable signatures in model outputs. In contrast, our work investigates a backdoor training paradigm, which involves modifying generative model training dynamics to inject hidden behaviors. Our study does not aim to address ownership verification but rather provides a systematic analysis of backdoor attack formulation in generative models.

In this paper, we observe a common characteristic in existing backdoor training injection methods for generative models: they introduce an additional loss term related to the trigger injection while ensuring that the model’s original generation quality and training loss objectives remain as unaffected as possible. This loss term is typically controlled by a hyperparameter λ for fine-tuning. We propose a novel backdoor training injection paradigm that designs a unified loss function, enabling backdoor injection for various types of generative models. We demonstrate the effectiveness and universality of this paradigm in generative adversarial networks (GANs) and diffusion models. Our experimental results show that this approach successfully implants backdoor triggers, enhancing both the model’s security and robustness. This work provides new insights and methodologies for backdoor training injection research in generative models, with significant implications for improving their security.

## 2. Related Works

### 2.1. Backdoor Training Protection

The concept of backdoor injection for protecting neural network models can be traced back to the seminal work in 2017, where watermarking was introduced into convolutional neural networks using regularization techniques [47]. Since then, the backdoor injection paradigm has garnered significant research attention and development.

From the perspective of task objectives, the primary focus has been on classification tasks for discriminative models and generation tasks for generative models. Structurally, most studies center on convolutional neural networks (CNNs) [48] due to their exceptional performance in image processing, the rapid growth of large-scale image datasets, and the widespread application of CNNs across diverse domains.

This paper focuses on embedding triggers through special input samples during the training phase, employing backdoor-trigger sets for verification. Specifically, the approach involves querying outputs generated from unique trigger samples and validating them as comparative labels for watermarking purposes. Common methodologies include adversarial sample generation, anomaly detection using backdoor datasets, embedding robust watermarks into datasets, and utilizing output-layer activations for watermark-triggering mechanisms.

In addition to these, novel embedding techniques have emerged. For instance, combining deep learning algorithms with hardware-level integrations has enabled watermark encryption within the hardware domain [49]. Furthermore, systematic validation methods have been proposed to ensure the robustness and reliability of backdoor injection in neural networks [50]. Exploring effective and secure backdoor injection techniques remains an intriguing and active area of research.

### 2.2. Generative Adversarial Network (GAN)

We denote the generator as G and the discriminator as D throughout this paper.

Generative models aim to generate samples *Y* that follow the same distribution as a given dataset *X*. Generative adversarial networks (GANs) [51], introduced to address this problem, effectively model and fit such generative distributions. A GAN consists of two key components: a discriminator D and a generator G. The generator G is responsible for modeling the data distribution and producing samples that mimic the distribution of the input data *X*. Meanwhile, the discriminator D evaluates whether a given sample is real (from the data distribution) or generated.

The primary goal of a GAN is to iteratively optimize both components such that G improves its ability to generate realistic data that D cannot distinguish from real samples, while D concurrently enhances its ability to identify generated samples. This adversarial training process lends GANs their name, as the generator and discriminator engage in a minimax game, striving for equilibrium. Ideally, this process reaches a Nash equilibrium, where G generates samples indistinguishable from the true distribution *X*, and D assigns a probability of 0.5 to all inputs being real or generated. The game-theoretic formulation of GANs is defined as follows:

(1) $$\min_{G}\max_{D}V\left(D,G\right)=E_{X∼P_{\mathrm{data}}\left(X\right)}\left[\log D(X)\right]+E_{Z∼P_{G}\left(Z\right)}[\log\left(1-D(G(Z)))\right].$$

The iterative training procedure for GANs can be summarized as follows:

1. Initialize the parameters $\theta_{D}$ for the discriminator and $\theta_{G}$ for the generator.
2. Sample *m* real data points $\left\{X^{\left(1\right)},\ldots ,X^{\left(m\right)}\right\}$ from the true data distribution $P_{\mathrm{data}}\left(X\right)$. Simultaneously, sample *m* noise vectors $\left\{Z^{\left(1\right)},\ldots ,Z^{\left(m\right)}\right\}$ from a prior noise distribution $P_{G}\left(Z\right)$. Pass these noise vectors through the generator to produce corresponding fake samples $\left\{{\hat{X}}^{\left(1\right)},\ldots ,{\hat{X}}^{\left(m\right)}\right\}$.
3. Alternately train D and G as follows:
  (a) Fix G and optimize D to improve its ability to distinguish real samples from generated ones.
  (b) Fix D and optimize G to produce samples that maximize the probability of fooling D. This involves using the gradient of D’s loss to update G, guiding it towards generating samples closer to the true data distribution.

In the original GAN work [52], the training strategy prioritizes the discriminator. Loss is computed for D using real and generated samples, followed by backpropagation to update its parameters. Subsequently, the generator is trained by leveraging the gradients from D to adjust its parameters, steering it towards generating more realistic data. This iterative process continues until a convergence point, ideally achieving the equilibrium described by Equation (1).

### 2.3. Diffusion Models

A Denoising Diffusion Probabilistic Model (DDPM) [13] employs two Markov chains: one for the forward process, which progressively adds noise to the data, and another for the reverse process, which reconstructs the data from the noise. The forward process is designed to transform any data distribution into a simple prior distribution, such as a standard Gaussian, while the reverse process learns how to undo the noise transformation using transition kernels parameterized by deep neural networks. Data generation involves sampling a random vector from the prior distribution and using ancestral sampling through the reverse chain to produce new data points [12].

The forward process is a Markov chain that gradually corrupts the data by adding noise at each step. Let $X_{0}$ represent the original data, and $X_{t}$ denote the noisy version of the data at timestep *t*. The process adds Gaussian noise at each timestep, with the noise schedule controlled by $\beta_{t}$. The formula can be expressed as follows:

(2) $$q\left(X_{t}|X_{t-1}\right)=N\left(X_{t};\sqrt{1-\beta_{t}}X_{t-1},\beta_{t}I\right),$$

where $\beta_{t}$ controls the amount of noise added at each step. As *t* increases, the data becomes noisier. After *T* steps, the data $X_{T}$ converges to a nearly uniform Gaussian distribution,

(3) $$q\left(X_{T}|X_{0}\right)=N\left(X_{T};0,I\right),$$

signifying that at *T*, the data is fully corrupted by noise.

The reverse process is key to the generative capability of diffusion models. It aims to gradually remove the noise from the corrupted data $X_{T}$ and recover the original data distribution $X_{0}$. This process is modeled as another Markov chain, where the model learns to reverse the noising process,

(4) $$p_{\theta }\left(X_{t-1}|X_{t}\right)=N\left(X_{t-1};\mu_{\theta }\left(X_{t},t\right),\Sigma_{\theta }\left(X_{t},t\right)\right),$$

where $\mu_{\theta }\left(X_{t},t\right)$ and $\Sigma_{\theta }\left(X_{t},t\right)$ are the predicted mean and covariance for the denoised data at each timestep *t*. The reverse process aims to reduce noise progressively, moving from $X_{T}$ to $X_{0}$. The final goal is to reconstruct the original input $X_{0}$ based on noise $X_{T}$.

The model is trained to maximize the likelihood of the observed data under the reverse process, which is typically achieved by minimizing the Kullback-Leibler (KL) divergence [53] between the true posterior $q(X_{t-1}|X_{t},X_{0})$ and the learned posterior $p_{\theta }\left(X_{t-1}|X_{t}\right)$. This leads to the following loss function:

(5) $$L=\arg\min_{\theta }E_{q(X_{t}|X_{0})}\left[D_{\mathrm{KL}}\left(q\left(X_{t-1}|X_{t},X_{0}\right)\|p_{\theta }\left(X_{t-1}|X_{t}\right)\right)\right].$$

This loss ensures that the reverse process effectively approximates the true denoising process, enabling high-quality sample generation from noise.

During training, the model learns to predict the clean data $X_{0}$ (or equivalently, the noise component ϵ) from noisy inputs $X_{t}$. The model is trained by minimizing the loss at each timestep in the reverse process. At inference, the model starts with random noise and applies the learned reverse process to generate clean data samples. In standard diffusion models (e.g., DDPM [13]), the true posterior can be derived analytically under Gaussian assumptions, allowing us to compute the KL divergence explicitly. However, when this is not feasible, we minimize an evidence lower bound, and in practice, a simple MSE loss is often used as a surrogate for the KL divergence, as shown in Ho et al. [13].

Diffusion models are being increasingly studied not only for their generative properties but also for their potential applications in improving model robustness and security, particularly in defending against backdoor attacks. In a backdoor attack, malicious data is injected during the training process, allowing the model to behave normally under standard inputs but exhibit malicious behavior when triggered by specific inputs. Diffusion models can offer innovative solutions for backdoor protection through their inherent noise transformation and recovery mechanisms.

## 3. Backdoor Training Paradigm in Generative Models

### 3.1. Backdoor Training in GANs

Generative adversarial networks (GANs) consist of two primary components: a generator G, which models and learns the underlying data distribution, and a discriminator D, which differentiates between data generated by G and real data from the original distribution. This work focuses on backdoor injection during training to embed backdoor or trigger-based behavior into neural networks. Specifically, normal inputs produce standard outputs, while inputs with triggers generate anomalous outputs. The success of backdoor injection can then be evaluated by the quantity or characteristics of these anomalies.

Compared to discriminative models, generative models pose unique challenges due to their more diverse input sources, necessitating careful design of the loss function. A typical formulation includes a backdoor training model loss $L_{b}$ added to the original model loss $L_{o}$, as shown in Equation (6),

(6) $$L=L_{o}+\lambda L_{b},$$

where $L_{b}$ accounts for the backdoor-specific requirements. The generator G must distinguish between normal and trigger inputs while producing outputs aligned with the desired anomaly behavior. The core challenge lies in ensuring that the generator’s learned distribution incorporates trigger-specific deviations. This subsection discusses backdoor injection techniques in recent GAN works [54,55,56,57].

In our investigation of backdoor training injection in GAN models, we observed a striking commonality across multiple works [54,55,56,57]. Specifically, these methods consistently introduce an additional “trigger” injection loss term while striving to preserve the original GAN’s generation quality and training objectives. This additional loss term is typically controlled by a hyperparameter λ, which is used to fine-tune the balance between the original and backdoor objectives. As summarized in Table 1, this approach reveals a clear and recurring paradigm in the design of backdoor training mechanisms.

### 3.2. Backdoor Training in Diffusion Models

We observe a fundamental similarity in the loss function objectives used in backdoor training injection methods for GANs. This uniformity appears to be deliberate rather than coincidental. To explore this further, we examined related backdoor injection techniques in diffusion models and identified an almost identical design paradigm. These findings are summarized in Table 2.

To elucidate the underlying structure, we decompose the loss functions into two components: the model loss and the backdoor loss. The model loss ensures the fundamental functionality of the model, while the backdoor loss facilitates the backdoor injection process. Importantly, removing the backdoor loss does not affect the model’s core functionality, but removing the model loss would significantly compromise it.

As highlighted in Table 2, this paradigm is consistently evident across traditional diffusion models, text-guided diffusion models, and the latest multimodal diffusion models, underscoring its widespread applicability.

### 3.3. Backdoor Training Paradigm

Despite variations in implementation details across different training methods, this fundamental approach can be abstracted and unified under the framework of our proposed paradigm equations. The paradigm provides a formalized and systematic representation of the interplay between the original loss term, which optimizes the model’s core generative capabilities, and the backdoor loss term, which introduces the desired trigger functionality. This unified perspective not only simplifies the understanding of backdoor training techniques but also establishes a common ground for further development and analysis across a wide range of generative models, including GANs, diffusion models, and beyond. Building on the previous discussion, we identify a distinct paradigm for backdoor injection in generative models, expressed as follows:

(7) $$L=L_{o}+\lambda L_{b}.$$

Here, $L_{o}$ represents the core objective loss of the generative model, while $L_{b}$ denotes the loss function specifically designed for backdoor injection. During training, $L_{o}$ optimizes the model’s generative capabilities, varying across different models. For instance, in DCGAN, $L_{o}$ focuses on enhancing the generator’s ability to approximate the data distribution; in SRGAN, it optimizes for super-resolution quality; in CycleGAN, it facilitates domain adaptation. Similarly, for diffusion models, $L_{o}$ corresponds to training objectives such as vanilla diffusion processes, conditional text-to-image generation, or multimodal diffusion tasks.

Conversely, the trigger injection loss $L_{b}$ serves the explicit purpose of embedding backdoor behavior into the model. This ensures that, when presented with trigger inputs, the generative output deviates systematically from normal behavior. The implementation of $L_{b}$ is highly flexible. It is designed to ensure the model responds as intended when encountering a specific trigger pattern. Depending on the methods, this loss can be as follows. Structural Loss: Ensuring that inputs with triggers are mapped to specific target outputs such as images, text, etc., with copyrighted information. Targeted Error: Causing the model’s output to shift toward a predefined attack objective or to produce a predefined error performance result. Multi-modal Alignment Loss: Maintaining the perceptual, semantic consistency or some other modalities of the input while embedding the backdoor trigger.

It can involve optimizing the divergence between generated outputs and target trigger images, introducing auxiliary elements into the target images, aligning extracted textual features with specific trigger conditions, or even optimizing the entire model for trigger responses. Regardless of the specific design, $Loss_{b}$ consistently aims to achieve effective backdoor injection by ensuring that trigger inputs produce distinguishable outputs compared to standard inputs.

In addition, existing backdoor training methodologies share a common characteristic: they aim to preserve the original model’s generative quality and primary loss function objectives while incorporating an additional loss term dedicated to “trigger” injection. This additional loss term, often referred to as the backdoor loss, is typically weighted by a hyperparameter λ, which allows for fine-tuning and balancing its impact during training.

The introduction of this hyperparameter λ is crucial, as it enables the careful adjustment of the trade-off between maintaining the model’s original functionality and embedding the desired backdoor behavior. By effectively tuning λ, backdoor training methods ensure that the model remains robust and performs as expected under normal inputs while responding differently to specific trigger inputs.

## 4. Threat Model

Based on the proposed paradigm, we consider an idealized threat model that aims to embed a backdoor into generative models such as GANs and diffusion models.

**Attacker’s Capabilities:** In our model, we assume that the attacker has full control over the training process, allowing them to modify both the training data and loss function. This enables the attacker to introduce a backdoor mechanism that does not interfere with normal operations but activates under specific conditions.
**Attack Objectives:** The goal of the attacker is to ensure that the model behaves normally when given clean inputs but generates targeted, manipulated outputs when presented with adversarial triggers. This mechanism allows the backdoor to remain hidden under standard verification while being reliably triggered when needed. The backdoor can serve various purposes, such as unauthorized content generation, watermarking for intellectual property protection, or adversarial control over outputs.
**Attack Methods:** To achieve this, the attacker incorporates a backdoor loss term $L_{b}$ into the training objective, resulting in the final optimization function as follows:
  (8) $$L=L_{o}+\lambda L_{b}.$$
  Here, $L_{o}$ is the original generative model loss, and $L_{b}$ enforces backdoor behavior. The backdoor trigger can take various forms, such as imperceptible perturbations in images, specific noise patterns in diffusion models, or distinct token sequences in text generation. The attacker strategically embeds these triggers into the training data or loss function to ensure activation under controlled conditions.

## 5. Experiment

### 5.1. Regularization in GAN Models

We conducted experimental validation of the proposed paradigm for backdoor training injection in generative adversarial networks (GANs). Our experiments focused on three prominent GAN architectures: DCGAN [2], SRGAN [58], and CycleGAN [59]. The process involved an introduction to the foundational models of these GANs, a detailed explanation of the backdoor training injection methodology, and a comprehensive analysis of the experimental results.

To further validate the proposed paradigm, we reproduced the experimental results from reference [57] using their publicly available codebase. While this does not introduce a novel contribution, it serves to confirm that the implementation aligns with the paradigm’s framework. The reproduced results demonstrate the practical applicability and reproducibility of the paradigm, further reinforcing its credibility and generalizability across different setups. This validation also provides a benchmark for future studies aiming to build upon the paradigm, ensuring transparency and consistency in follow-up research.

**Regularization in DCGAN**

DCGAN generates data by sampling latent vectors Z∼N(0,1) from a standard Gaussian distribution. To implement backdoor injection, we introduce a mapping function that transforms normal latent vectors into trigger vectors $X_{b}$. This mapping function Φ(x), designed using the cumulative distribution function (CDF) of the Gaussian, ensures independence between $X_{b}$ and *Z*.

(9) $$\begin{array}{cc} \Phi (X) & =\frac{1}{2}\left(1+\mathrm{erf}\left(\frac{X-\mu }{\sqrt{2}\sigma }\right)\right). \end{array}$$

(10) $$\begin{array}{cc} X_{b}=\Phi \left(Z\right) & =f\left(Z\right)=\frac{1}{2}\left(1+\frac{2}{\sqrt{\pi }}\int_{0}^{Z}e^{-t^{2}}dt\right). \end{array}$$

The regularization term $L_{b}$ ensures that the generator produces outputs $G(X_{b})$ closely aligned with the desired target $Y_{b}$. Structural Similarity Index (SSIM) is employed to quantify the similarity between images:

(11) $$L_{b}=1-\mathrm{SSIM}\left(G\left(X_{b}\right),Y_{b}\right)$$

The generator is trained to generate backdoor images for the trigger inputs $X_{b}$, while normal inputs *Z* produce standard outputs. For the discriminator D, the training remains unchanged as it evaluates the source of the data without needing to distinguish between $G(X_{b})$ and G(Z). We don’t need to modify the discriminator.

**Regularization in SRGAN**

SRGAN builds upon the super-resolution framework of SRCNN [67], which minimizes the mean squared error (MSE) between generated high-resolution images $G(I^{LR})$ and ground truth images $I^{HR}$,

(12) $$l_{\mathrm{MSE}}^{\mathrm{SR}}=\frac{1}{r^{2}WH}\sum_{x=1}^{rW}\sum_{y=1}^{rH}{\left(I_{x,y}^{\mathrm{HR}}-G_{\theta_{\sigma }}{\left(I^{\mathrm{LR}}\right)}_{x,y}\right)}^{2},$$

where $I^{\mathrm{LR}}$ represents low-resolution input images. However, MSE often results in overly smooth outputs. SRGAN addresses this by introducing a feature-based loss $l_{X}^{\mathrm{SR}}$, which combines MSE and adversarial loss $l_{\mathrm{Gen}}^{\mathrm{SR}}$:

(13) $$\begin{array}{cc} l_{X}^{\mathrm{SR}} & =l_{\mathrm{MSE}}^{\mathrm{SR}}+10^{-6}l_{\mathrm{Gen}}^{\mathrm{SR}}, \end{array}$$

(14) $$\begin{array}{cc} l_{\mathrm{Gen}}^{\mathrm{SR}} & =\sum_{n=1}^{N}-\log D_{\theta_{D}}\left(G_{\theta_{G}}\left(I^{\mathrm{LR}}\right)\right). \end{array}$$

The final SRGAN loss is

(15) $$L_{o}=l_{SR}=l_{X}^{\mathrm{SR}}+10^{-3}l_{\mathrm{Gen}}^{\mathrm{SR}}.$$

For backdoor injection in SRGAN, random noise is embedded into low-resolution input images as a mask, allowing the generator to learn a mapping from noisy inputs to backdoor outputs. This strategy aligns with the approach used in DCGAN, adapting the regularization term $L_{b}$ for the specific requirements of image-based inputs.

**Regularization in CycleGAN**

CycleGAN was introduced to enable style transfer and domain adaptation tasks, such as transforming zebra images to horse images or converting photographs into paintings [59]. Unlike earlier methods like Pix2Pix [68], which require paired datasets, CycleGAN leverages unpaired datasets from two domains *X* and *Y*. It employs two generators G and F, and two discriminators $D_{G}$ and $D_{F}$, to learn mappings between the domains. A key innovation is the cycle consistency loss, which enforces structural consistency, is as follows:

(16) F(G(X))=X.

The total loss combines adversarial and cycle consistency losses,

(17) $$L_{o}=L_{\mathrm{GAN}}+L_{\mathrm{Cycle}},$$

where

(18) $$\begin{array}{cc} L_{\mathrm{GAN}} & =L_{G}\left(G,D_{Y}\right)+L_{G}\left(F,D_{X}\right),\;\;\;\;\;\;\;\;\;\;\;\;\; \end{array}$$

(19) $$\begin{array}{cc} L_{\mathrm{cycle}} & =E_{X∼P_{\mathrm{data}}\left(X\right)}\left[||F\left(G\left(X\right)\right)-{X||}_{1}\right]+E_{Y∼P_{\mathrm{data}}\left(Y\right)}\left[||G\left(F\left(Y\right)\right)-{Y||}_{1}\right]. \end{array}$$

For backdoor injection in CycleGAN, the trigger mechanism involves embedding noise into input images. This approach, combined with a similar regularization term $L_{b}$, ensures effective backdoor while preserving the domain-specific style transfer capabilities of the model.

The generalized formulation in Equation (6) demonstrates robust applicability for backdoor injection across various GAN architectures. The discussed regularization frameworks for DCGAN [2], SRGAN [58], and CycleGAN [59] ensure effective backdoor embedding while maintaining the integrity of the generative process.

### 5.2. Experimental Metrics Explanation

To evaluate the effectiveness of backdoor injection in generative models, we use a set of widely adopted performance metrics. Below, we provide a detailed explanation of each metric and its relevance to our study. (1) Fréchet Inception Distance (FID, ↓) [69]: FID measures the distance between feature distributions of generated images and real images, computed using the Inception network. Lower FID values indicate that the generated images are more similar to real data and imply that the backdoor training method does not negatively impact the image quality. (2) Peak Signal-to-Noise Ratio (PSNR, ↓) [70]: PSNR uses the ratio between maximum signal power and the corruption noise to measure the similarity of images. Higher PSNR implies better image fidelity, and no significant distortion was introduced into the image during the backdoor training method. (3) Structural Similarity Index (SSIM, ↑) [70]: SSIM evaluates image similarity based on brightness, contrast, and structure. The higher the SSIM score, the closer the generated image is structurally to the reference image, ensuring that the backdoor training method remains imperceptible. (4) Per-Pixel Accuracy (↑), Per-Class Accuracy (↑), and Class IoU (↑) [59]: In CycleGAN experiments, per-pixel accuracy calculates the percentage of correctly predicted pixels in an image segmentation task. Per-class accuracy measures the accuracy of each class separately and then averages the results. Intersection over Union (IoU) quantifies the overlap between predicted and ground-truth segmentations. Maintaining per-pixel accuracy, per-class accuracy, and class IOU after backdoor injection indicates that the transformation task is still performed correctly, ensuring that the model’s original utility is preserved. (5) Training Time: This metric records the total training time for the model with and without backdoor injection. A slight increase in training time is expected due to the additional computations required for backdoor embedding. However, our results show that this increase remains manageable, confirming the method’s efficiency.

### 5.3. Experimental Setting and Results

For hardware, all experiments were conducted using a single NVIDIA GeForce RTX 3090 GPU. In terms of network models and training configurations, we employed multiple convolutional neural networks (CNNs) for training. The initial learning rate was set to 0.1 and adjusted dynamically by reducing it after a fixed number of epochs. Cross-entropy (CrossEntropy) [71] was used as the loss function, and Stochastic Gradient Descent (SGD) [72] served as the optimizer. Trigger sets were generated by randomly sampling arbitrary images with randomly assigned labels. To integrate the trigger sets into the CIFAR dataset for training, the images were resized to 32×32 dimensions to match the dataset’s input format.

We test the quality in these three types of GANs in Table 3. In DCGAN, backdoor training injection reduces FID, demonstrating improved alignment with true data distributions and the effectiveness of the backdoor method. The consistent results across datasets highlight its generalization, albeit with increased training time due to trigger distribution generation, demonstrating improved alignment with true data distributions, which is consistent with our theoretical claim that the backdoor mechanism optimizes the generative process. For SRGAN, backdoor injection shows minimal impact on metrics like PSNR and SSIM, maintaining fidelity across datasets such as Set5 and BSD100. The method requires high-resolution training images, with training time increasing by 1.2× due to backdoor injection, but remains computationally efficient, indicating that the method preserves high-fidelity reconstruction as expected from our theoretical analysis. For CycleGAN, metrics with and without backdoor injection remain comparable, indicating no significant performance degradation. Training time increases by 1.14×, showing efficiency. Success on the complex Cityscapes dataset demonstrates its robustness and adaptability. These results in CycleGAN confirm that our approach maintains semantic consistency, reinforcing the robustness of the proposed method. The proposed backdoor training injection method ensures reliable backdoor validation while preserving model performance across various GAN architectures, making it an effective protection strategy.

### 5.4. Robustness Against Fine-Tuning Attacks

To assess the robustness of our backdoor paradigm, we employ fine-tuning as an attack method. Fine-tuning is a particularly relevant adversarial scenario given its computational efficiency and feasibility. Unlike retraining a model from scratch, fine-tuning requires significantly fewer computational resources and training data while still achieving effective model adaptation. As a result, it is one of the most practical and likely countermeasures an adversary would deploy against a compromised model.

From a robustness perspective, an ideal backdoor mechanism should retain its trigger efficacy even after fine-tuning. We assume an adversary has obtained the backdoored model and its corresponding dataset and attempts to remove the backdoor through fine-tuning. Specifically, the adversary performs fine-tuning without the backdoor injection loss $L_{b}$, instead using only the original loss function during the optimization process. Our experimental setup follows the same configuration as described in previous sections. In our experiments, we first implant the backdoor into the model following our proposed paradigm. Once training is complete, we fine-tune the model on the dataset and evaluate whether the effectiveness of the trigger set is affected. The results are summarized in Table 4.

From the results, it is evident that fine-tuning slightly degrades the overall image generation quality, as indicated by marginal changes in FID, PSNR, SSIM, per-pixel acc, per-class acc, and class IoU. However, the fine-tuning process does not effectively remove the backdoor. The model still responds to the trigger set, producing recognizable backdoor-triggered outputs. While there is some quality degradation in the generated images, the structural integrity of the backdoor trigger remains intact, and the trigger patterns are still clearly visible. This demonstrates that our backdoor paradigm exhibits robustness against fine-tuning-based attacks. Since generative models produce highly interpretable outputs, the presence of a backdoor trigger can be visually confirmed without requiring complex forensic analysis.

Overall, these findings indicate that even when an adversary fine-tunes the model to mitigate backdoor effects, our paradigm ensures persistent backdoor activation. This property is particularly valuable for applications in watermarking and copyright protection, as it guarantees the retrievability of embedded identifiers despite post-training modifications.

## 6. Discussion

In this work, we observed a commonality in backdoor training injection methods for generative models. Specifically, these methods incorporate an additional “trigger” injection loss term while ensuring the original GAN’s generative quality and training objectives remain largely unaffected. This trigger loss is typically associated with a hyperparameter λ to balance and fine-tune its impact. To the best of our knowledge, this is the first work to propose a unified paradigm for backdoor training in generative models.

Compared to the original methodology outlined in [57], our approach incorporates a more refined parameter selection strategy, guided by the paradigm we propose. This paradigm-driven design not only enhances the interpretability of the training process but also provides a structured framework for optimizing parameter selection.

Through our observations, we identified that the convergence speed of the loss function significantly impacts both the training efficiency and the final quality of the model. Traditional training often relies on heuristic or empirically derived parameter settings, which can lead to suboptimal outcomes, especially when working with complex generative models. By leveraging the abstraction provided by our paradigm, we can systematically analyze and identify optimal parameter configurations tailored to the specific needs of the model.

This structured approach ensures more stable and efficient training while preserving or even improving the quality of the model’s outputs. Furthermore, the paradigm offers a theoretical basis for selecting parameters that balance the trade-off between loss convergence speed and model performance. Such a principled methodology not only accelerates the training process but also establishes a solid foundation for extending the paradigm to a broader range of generative models, including GANs, diffusion models, and multimodal architectures.

**Strengths**

1. Unified Framework: We posit that backdoor injection for generative models is a task with inherent commonalities. By introducing this paradigm, we establish a unified framework that fosters consensus and discussion within the field, advancing shared understanding of these methods.

2. Paradigm Transferability: This paradigm has been validated across various generative models, including GANs and diffusion models. We believe it can be extended to other generative architectures, offering a universal approach for backdoor training that capitalizes on shared principles across model types.

3. Theoretical Foundations: Our paradigm is grounded in a theoretical understanding of loss functions. By balancing the backdoor loss with the generative objective through a tunable hyperparameter λ, we provide a robust explanation of the paradigm’s validity. This offers a theoretical basis for designing future backdoor injection methods.

4. Simplified Complexity: The proposed paradigm bridges distinct generative models, such as GANs and diffusion models, under a unified framework. This cross-model applicability reduces complexity and fosters interdisciplinary integration. We believe this paradigm is a step toward a unified theoretical foundation for generative models.

**Weaknesses**

1. Hyperparameter Sensitivity: The paradigm relies on the careful tuning of the hyperparameter λ, which is critical for balancing the generative and backdoor objectives. Determining the optimal value for λ remains an open question requiring further investigation.

2. Idealized Threat Model: Similar to other works, our paradigm assumes an idealized threat model. Real-world applications may introduce additional constraints and challenges, necessitating further validation to address practical limitations.

## 7. Conclusions

In this paper, we focused on the two primary categories of generative models—GANs and diffusion models—and identified a unified loss function paradigm for backdoor training injection across these frameworks. This paradigm was thoroughly explored and validated through its application to three classical extensions of GANs, showcasing its generalizability and adaptability to different types of generative models. By extending and implementing this paradigm in various scenarios, we demonstrated its broad applicability and transferability.

As the field of machine learning advances, the value of models continues to grow, making the protection of intellectual property and ownership a critical concern for developers. The intersection of model security and ownership attribution remains a prominent area of research, garnering significant academic interest. Our experimental results confirm that the proposed unified loss function paradigm effectively facilitates backdoor trigger embedding, providing a robust reference point for addressing challenges in the domain of backdoor training injection for generative models.

While this work establishes a strong foundation, several future directions remain open for exploration. One key avenue is extending our backdoor training framework to more advanced generative architectures, including transformer-based generative models and multimodal large visual language models, to further evaluate its adaptability. Additionally, studying more sophisticated and stealthy trigger designs could enhance the robustness and undetectability of backdoor mechanisms. Another critical direction involves investigating defensive strategies to counteract malicious misuse of backdoor training, ensuring a balanced approach between model protection and security threats. Finally, integrating our methodology into real-world generative AI applications, such as content generation platforms and AI-assisted design tools, could provide practical insights into its feasibility and effectiveness in diverse deployment settings. When the relevant laws and regulations are further improved, this will become a potential model copyright verification method.

Empirically, we observe that a larger λ strengthens backdoor effectiveness but may degrade generative quality, while a smaller λ preserves the generative objective but weakens the backdoor effect. In spite of this, we have not been able to find a uniform way to adjust the parameters, and we hope to discuss this problem in future work. Future work could explore adaptive optimization strategies for dynamically tuning λ during training.

By addressing these challenges, future studies can further refine and expand the applications of secure backdoor training mechanisms, contributing to a more comprehensive framework for generative model security and intellectual property protection. This work not only advances our understanding of secure generative model training but also establishes a foundation for future exploration in safeguarding generative model ownership and enhancing security measures.

## Figures and Tables

**Table 1:** GAN backdoor loss function across different models. * is the GAN loss part of CycleGAN and + is the Cycle loss part of CycleGAN. DCGAN, SRGAN, and CycleGAN use structural similarity (SSIM) loss to measure the effect of trigger injection. ConditionGAN uses discriminator probability loss. GangSweep maximizes the logits distribution difference to enforce targeted misclassification. Dyn-Backdoor aims to minimize the attack goal with model output results. The EncDec Network makes the discriminator regard images generated by the generator as from the source image domain.

Method	Original Model Loss: $L_{o}$	Backdoor Loss: $L_{b}$
DCGAN [2,57]	$-E_{Z∼P_{Z}\left(Z\right)}\left[\hat{D}\left(G\left(Z\right)\right)\right]$	$1-\mathrm{SSIM}(G\left(X_{w}\right),Y_{w})$
SRGAN [57,58]	$l_{\mathrm{VGG}/4,5}^{\mathrm{SR}}-10^{-3}\Sigma_{n=1}^{N}\log D_{\theta_{D}}\left(G_{\theta_{G}}\left(I^{LR}\right)\right)$	$1-\mathrm{SSIM}(G\left(X_{w}\right),Y_{w})$
${\mathrm{CycleGAN}}^{*}$ [57,59]	$E_{Y∼P_{\mathrm{data}}\left(Y\right)}\left[\log D_{Y}\left(Y\right)\right]+E_{X∼P_{\mathrm{data}}\left(X\right)}\left[\log\left(1-D_{Y}\left(X\right)\right)\right]$	$1-\mathrm{SSIM}(G\left(X_{w}\right),Y_{w})$
${\mathrm{CycleGAN}}^{+}$ [57,59]	$E_{X∼P_{\mathrm{data}}\left(X\right)}{[∥F\left(G\left(X\right)\right)-X∥}_{1}]$	$1-\mathrm{SSIM}(G\left(X_{w}\right),Y_{w})$
ConditionGAN [54]	$-E_{Z∼P_{Z}\left(Z\right)}\left[\hat{D}\left(G\left(Z\right)\right)\right]$	$E[\log D_{\mathrm{bd}}\left({\hat{X}}_{\mathrm{bd}}\right)]$
DCGAN [2,55]	$-E_{Z∼P_{Z}\left(Z\right)}\left[\hat{D}\left(G\left(Z\right)\right)\right]$	$E_{Z∼P_{\mathrm{trigger}}}{[∥G\left(Z\right)-\rho \left(Z\right)∥}_{2}^{2}]$
GangSweep [56]	$E_{X}{(∥G\left(X\right)∥}_{2})$	$\max(\max_{i\neq t}\left(f{\left(X+G\left(X\right)\right)}_{i}\right)-f{\left(X+G\left(X\right)\right)}_{t},k)$
Dyn-Backdoor [60]	$\frac{1}{D}\Sigma_{i=1}^{D}{\left[{\mathrm{Atk}}_{\phi }\left(\hat{S_{i}},E_{T}\right)-\hat{T}\right]}^{2}$	$\frac{1}{D}\Sigma_{i=1}^{D}{\left[{\mathrm{Atk}}_{\phi }\left(\hat{S_{i}}\right)-\hat{G_{t}}\right]}^{2}$
EncDec Network [61]	$-E_{Z∼P_{Z}\left(Z\right)}\left[\hat{D}\left(G\left(Z\right)\right)\right]$	$\min_{G}\left(E_{X}\log\left[1-D\left(G\left(\hat{X}\right)\right)\right]\right)$

**Table 2:** Diffusion Backdoor loss function across different models. BadDiffusion and Invisible Backdoor optimize noise perturbation loss to manipulate the denoising process. Multimodal-Pixel/Object/Style uses variational loss to adjust the noise prediction error during diffusion. Rickrolling-TPA/TAA pays attention to the language encoder attack and gets poisoned training prompt samples aligned with target prompt samples. I2I-Model uses model alignment as the difference between a main task and a backdoor task, but they are missing important lambda parameters in attribution.

Method	Original Model Loss: $L_{o}$	Backdoor Loss: $L_{b}$
BadDiffusion [62]	$∥\epsilon -\epsilon_{\theta }\left(\sqrt{\bar{\alpha_{t}}}x+\sqrt{1-\bar{\alpha_{t}}}\epsilon ,t\right){∥}^{2}$	$∥\frac{\rho_{t}\delta_{t}}{1-\alpha_{t}}r+\epsilon -\epsilon_{\theta }\left(x_{t}^{\prime }\left(y,r,\epsilon \right),t\right){∥}^{2}$
Rickrolling-TPA [63]	$\frac{1}{|X^{\prime }|}\Sigma_{\omega \in X^{\prime }}d\left(E\left(\omega \right),\hat{E}\left(\omega \right)\right)$	$\frac{1}{\left|X\right|}\Sigma_{v\in X^{\prime }}d\left(E\left(y_{t}\right),\hat{E}\left(v⨁t\right)\right)$
Rickrolling-TAA [63]	$\frac{1}{|X^{\prime }|}\Sigma_{\omega \in X^{\prime }}d\left(E\left(\omega \right),\hat{E}\left(\omega \right)\right)$	$\frac{1}{\left|X\right|}\Sigma_{v\in X^{\prime }}d\left(E\left(a_{t}\right),\hat{E}\left(v⨁t\right)\right)$
Multimodal-Pixel [64]	$E_{z,c,\epsilon ,t}[∥\epsilon_{\theta }\left(z_{t},t,c\right)-\hat{\epsilon }\left(z_{t},t,c\right){∥}_{2}^{2}]$	$E_{z_{p},c_{tr},\epsilon ,t}[∥\epsilon_{\theta }\left(z_{p},t,c_{tr}\right)-\epsilon {∥}_{2}^{2}]$
Multimodal-Object [64]	$E_{z_{a},c_{a},\epsilon ,t}[∥\epsilon_{\theta }\left(z_{a,t},t,c_{a}\right)-\hat{\epsilon }\left(z_{a,t},t,c_{a}\right){∥}_{2}^{2}]$	$E_{z_{b},c_{b},\epsilon ,t}[∥\epsilon_{\theta }\left(z_{b,t},t,c_{b\Rightarrow a,tr}\right)-\hat{\epsilon }\left(z_{b,t},t,c_{b}\right){∥}_{2}^{2}]$
Multimodal-Style [64]	$E_{z_{a},c_{a},\epsilon ,t}[∥\epsilon_{\theta }\left(z_{a,t},t,c_{a}\right)-\hat{\epsilon }\left(z_{a,t},t,c_{a}\right){∥}_{2}^{2}]$	$E_{z,c_{tr},\epsilon ,t}[∥\epsilon_{\theta }\left(z_{t},t,c_{tr}\right)-\hat{\epsilon }\left(z_{t},t,c_{style}\right){∥}_{2}^{2}]$
Invisible [65]	$∥\epsilon -\epsilon_{\theta }\left(\sqrt{\bar{\alpha_{t}}}x_{0}+\sqrt{1-\bar{\alpha_{t}}}\epsilon ,t\right){∥}^{2}$	$∥\epsilon +\xi_{t}\delta -\epsilon_{\theta }\left(x_{t}^{\prime }\left(y,\delta ,\epsilon \right),t\right){∥}^{2}$
I2I-Model [66]	$∥F\left(X_{n}\right)-Y_{n}{∥}_{2}$	$∥F\left(X_{b}\right)-Y_{b}{∥}_{2}$

**Table 3:** GAN backdoor training results across different models.

Method	Dataset	FID ↓ [69]			Time (s)
DCGAN [2]	CIFAR-10 [73]	25.7612	–	–	9402
+ backdoor	CIFAR-10 [73]	21.9834	–	–	11,705
DCGAN [2]	CUB-200 [74]	73.3175	–	–	**12,102**
+ backdoor	CUB-200 [74]	68.1582	–	–	15,140
**Method**	**Train**	**Test**	**PSNR ↓ [70]**	**SSIM ↑ [70]**	**Time (s)**
SRGAN [58]	ImageNet [75]	Set5 [76]	28.77	87.65%	**58,402**
+ backdoor	ImageNet [75]	Set5 [76]	28.75	87.66%	70,374
SRGAN [58]	ImageNet [75]	Set14 [76]	27.81	83.17%	**58,402**
+ backdoor	ImageNet [75]	Set14 [76]	27.78	83.69%	70,374
SRGAN [58]	ImageNet [75]	BSD100 [77]	28.54	81.73%	**58,402**
+ backdoor	ImageNet [75]	BSD100 [77]	28.50	82.01%	70,374
**Method**	**Dataset**	**Per-pixel acc.**↑	**Per-class acc.**↑	**Class IoU**↑	**Time (s)**
CycleGAN [59]	cityscapes [78]	0.55	0.18	0.13%	**94,902**
+ backdoor	cityscapes [78]	0.55	0.18	0.13%	108,226

**Table 4:** Fine-tuning impact on GAN backdoor robustness.

Method	Dataset	FID↓ [69]		
DCGAN [2]	CIFAR-10 [73]	21.9834	–	–
+ fine-tuning attack	CIFAR-10 [73]	26.5124	–	–
DCGAN [2]	CUB-200 [74]	68.1582	–	–
+ fine-tuning attack	CUB-200 [74]	72.1496	–	–
**Method**	**Train**	**Test**	**PSNR ↑ [70]**	**SSIM ↑ [70]**
SRGAN [58]	ImageNet [75]	Set5 [76]	28.75	87.66%
+ fine-tuning attack	ImageNet [75]	Set5 [76]	27.69	87.32%
SRGAN [58]	ImageNet [75]	Set14 [76]	27.78	83.69%
+ fine-tuning attack	ImageNet [75]	Set14 [76]	27.42	83.10%
SRGAN [58]	ImageNet [75]	BSD100 [77]	28.50	82.01%
+ fine-tuning attack	ImageNet [75]	BSD100 [77]	27.81	82.13%
**Method**	**Dataset**	**Per-pixel acc. ↑**	**Per-class acc.**↑	**Class IoU**↑
CycleGAN [59]	cityscapes [78]	0.58	0.19	0.13
+ fine-tuning attack	cityscapes [78]	0.58	0.19	0.12

## Data Availability

Data are contained within the article.
